# Delayed diagnosis of pediatric brucellosis: a case study and literature review

**DOI:** 10.1097/MS9.0000000000003278

**Published:** 2025-04-10

**Authors:** Chunsong Pang, Lina Liu, Ting Liu, Nianzhu Zhang, Lifen Zhao

**Affiliations:** Department of Laboratory Medicine, The Second Hospital of Dalian Medical University, Dalian, Liaoning, China

**Keywords:** brucellosis, children, delayed diagnosis, misdiagnosis, underdiagnosis

## Abstract

**Introduction and importance:**

Brucellosis is globally acknowledged as the predominant zoonotic infection afflicting humans. Misdiagnosis and underdiagnosis pose significant challenges in the diagnosis of brucellosis due to the potential risk of overlooking or misinterpreting symptoms.

**Case presentation:**

This article reports a case of a 10-year-old girl with brucellosis, presenting with fever, rash on both lower limbs, and pain in the right ankle joint. The patient underwent 10 days of inaccurate treatment at a local hospital without improvement, and then sought treatment at a provincial hospital. Considering that it was the peak season for Mycoplasma pneumonia infection, she received intravenous cefotaxime combined with oral azithromycin for anti-infection treatment after admission. Six days later, blood cultures showed positive for *Brucella*, and it was learned that the child’s family raised sheep. She was ultimately diagnosed with brucellosis.

**Clinical discussion:**

In this case, if medical staff had been fully aware of the relevant issues and promptly conducted corresponding laboratory tests, such as the tube agglutination test (SAT) or the colloidal gold immunochromatographic assay, the patient might have received effective treatment earlier.

**Conclusion:**

Brucellosis often has nonspecific symptoms that can lead to misdiagnosis or delays in diagnosis. However, early intervention can reduce complications, shorten treatment time, and improve outcomes. Therefore, developing effective diagnostic tests for brucellosis is crucial for timely detection and management. Additionally, healthcare professionals need a thorough understanding of the disease’s clinical signs and diagnostic challenges to ensure accurate and prompt diagnoses.

## Introduction

Human brucellosis is an often persistent illness mimicking flu-like symptoms, with nonspecific clinical manifestations. This zoonotic disease is still common in many developing countries, especially in areas with widespread agriculture. The main mode of transmission is frequently associated with the consumption of unpasteurized dairy products^[^1^]^. Additionally, infection can be contracted through direct contact with infected animals, and there have been reported instances of individuals acquiring the disease following interactions with wildlife^[^2^]^.

The global prevalence of human brucellosis has significantly changed in the past decade compared to 2006^[^3^]^. The epidemic now spans at least 97 countries, including an additional 18 in Europe, 12 in the Americas, 9 in Asia, and 4 in Africa. The Eastern Mediterranean region has the highest reported incidence of brucellosis. Asia bears the highest burden of human brucellosis globally. In China, the incidence rate has witnessed a significant increase from 0.0281/100 000 in 1993 to 5.0553/100 000 in 2021^[^4^]^.

**HIGHLIGHTS**

- Human brucellosis is a persistent illness with nonspecific clinical symptoms. Often mimicking flu-like symptoms, this zoonotic disease is still common in many developing countries, especially in areas with widespread agriculture.
- Asia bears the highest burden of human brucellosis globally.
- Misdiagnosis and underdiagnosis pose significant challenges in the diagnosis of brucellosis due to the potential risk of overlooking or misinterpreting symptoms.
- This article presents a case study of a 10-year-old female patient diagnosed with Brucellosis, who exhibited symptoms including fever, bilateral lower limb rash, and pain in the right ankle joint. Following 16 days of diagnosis and treatment, the definitive diagnosis of the disease was established. Therefore, medical professionals need to have a thorough understanding of the clinical manifestations and diagnostic challenges of the disease in order to make accurate and timely diagnoses.

Misdiagnosis and underdiagnosis pose significant challenges in the diagnosis of brucellosis due to the potential risk of overlooking or misinterpreting symptoms. The nonspecific nature of symptoms, such as fever, fatigue, and joint pain, can resemble those associated with various other illnesses, potentially leading to delayed initiation of appropriate treatment.

It has previously been observed that 62.5% of brucellosis patients are misdiagnosed during their initial diagnosis^[^5^]^. Another study also reported that over half of the brucellosis patients were initially misdiagnosed with other diseases^[^6^]^. Recently, Qin *et al*^[^7^]^ reported a case of brucellosis patient who was misdiagnosed due to symptoms similar to COVID-19 or flu and delayed treatment during strict COVID-19 prevention and control period. Despite its low mortality rate, brucellosis can result in significant debilitation or impairment^[^8^]^. Therefore, prompt diagnosis and treatment are essential for preventing the chronic progression of the disease.

We are reporting a case of a child diagnosed with brucellosis after 16 days after the symptoms appeared. This study aims to contribute to Pediatric brucellosis literature, epidemiological, clinical features, laboratory findings, and treatment.

## Case report

On July 13, 2024, a 10-year-old girl came for a medical consultation due to fever, a rash on both lower limbs, and pain in the right ankle joint. The patient had developed a fever 10 days prior, with a maximum temperature of 39°C, accompanied by chills. Despite taking oral antipyretics and undergoing physical cooling, the fever persisted, recurring about three times daily. Oral amoxicillin capsules (0.25 g each, three times a day) proved ineffective after 2 days, leading to the development of scattered hemorrhagic rashes on both lower limbs and pain in the right ankle joint. Following hospitalization at a local facility, it was noted that the peak of the fever had decreased compared to before but still recurred, the rash had slightly reduced, and there was no significant improvement in the right ankle joint. Consequently, the patient was referred to our hospital for further diagnosis and treatment. The blood routine results from XXX Hospital on July 11, 2024, indicated the following: CRP 22.8 mg/L, WBC 4.687 × 10^9^/L, N% 39%, L% 58.5%, M% 2.3%, N# 1.9 × 10^9^/L, HGB 108 g/L, PLT 133 × 10^9^/L. Additionally, the serum amyloid A level was 95.78 mg/L; lactate dehydrogenase level was 677 U/L; alanine aminotransferase level was 42 U/L; and aspartate aminotransferase level was 94 U/L. Kidney function and myocardial enzymes are within normal ranges. However, the serum ferritin level was found to be over 1000 ng/ml. Tests for various viral antibodies all returned negative results. The routine urine analysis is normal. The specific results are detailed in Table 1. Chest X-ray suggests: increased and blurred lung markings in both lungs, and ultrasound indicated splenomegaly (oblique diameter 14.5 cm). In a follow-up examination on July 13, 2024, CRP levels had decreased to 14.65 mg/L, but other indicators remained unchanged.

**Table 1:** Laboratory results

	Lab tests	Result	Reference range
Blood test	C-reactive protein (CRP)	22.8 mg/L	0–10 mg/L
	White blood cells (WBC)	4.687 × 10^9^/L	3.50–9.50 × 10^9^/L
	Percentage of neutrophils (N%)	39%	40.00%–75.00%
	Percentage of lymphocytes (L%)	58.50%	20.00%–50.00%
	Percentage of monocytes (M%)	2.30%	3.00%–10.00%
	Absolute neutrophil count (N #)	1.9 × 10^9^/L	1.80–6.30 × 10^9^/L
	Hemoglobin (HGB)	108 g/L	115–150 g/L
	Blood platelets (PLT)	133 × 10^9^/L	125–350 × 10^9^/L
	Serum amyloid A (SAA)	95.78 mg/L	0–10 mg/L
	Alanine aminotransferase (ALT)	42 U/L	7–40 U/L
	Aspartate aminotransferase (AST)	94 U/L	13–35 U/L
	AST/ALT(AST/ALT)	2.23	0.5–1.5
	Lactate dehydrogenase (LDH)	677 U/L	120–250 U/L
	α-hydroxybutyrate dehydrogenase (α-HBDH)	500.31 U/L	72–182 U/L
	Creatine kinase (CK)	17.54 U/L	40–200 U/L
	Creatine kinase isoenzyme MB (CK-MB)	20.34 U/L	0–24 U/L
	Urea	3.53 mmol/L	2.6–7.5 mmol/L
	Creatinine (Cr)	31.35 μmol/L	41–73 μmol/L
	Uric acid (UA)	236.50 μmol/L	155–357 μmol/L
	Serum ferritin (FER)	1535.54 ng/ml	10–291 ng/ml
	Nucleic acid of influenza A virus	Negative (−)	Negative (−)
	Nucleic acid of influenza B virus	Negative (−)	Negative (−)
	Respiratory syncytial virus	>40	CT >40 was considered negative
	Adenovirus	>40	CT >40 was considered negative
	Parainfluenza virus type I	>40	CT >40 was considered negative
	Parainfluenza virus type II	>40	CT >40 was considered negative
	Parainfluenza virus type III	>40	CT >40 was considered negative
	Human metapneumovirus	>40	CT >40 was considered negative
	Mycoplasma pneumonia	>40	CT >40 was considered negative
Urine test	Glucose (GLU)	Norm mmol/L	-
	Bilirubin (BIL)	Neg µmol/L	-
	Urobilinogen (URO)	Norm µmol/L	Normal ~ +/−
	PH value (PH)	8	4.5–8.0
	Specific gravity (SG)	1.018	1.003–1.030
	Occult Blood (BLD)	Neg mg/L	-
	Nitrite (NIT)	Neg	-
	Ketone body (KET)	Neg mmol/L	-
	Urinary protein (PRO.)	Neg g/L	-
	White blood cells (LEU)	Neg/µl	-
	Urinary red blood cells (RBC/HP)	0.60/HP	0.00–2.27 /HP
	Urine white blood cells (WBC/HP)	0.90/HP	0.00–2.73 /HP
	Pus cell (U_PC)	0/µl	0–9/µl

Upon admission on July 13, 2024, the physical examination revealed scattered hemorrhagic rashes on both lower limbs. These rashes presented as a mixed distribution of petechiae and ecchymosis, slightly raised above the skin surface, and did not blanch upon pressure. The throat appeared congested, with mild enlargement of both tonsils. The liver was palpated 2 cm below the costal margin, and the spleen was palpated 3 cm below the costal margin, with no tenderness noted upon palpation. The joints of the limbs were normal, except for swelling in the right ankle joint, which had full range of motion. All other examination results were within normal limits. Given that it was the season for an outbreak of Mycoplasma pneumonia, intravenous cefotaxime combined with oral azithromycin was administered for anti-infection treatment after admission. Simultaneously, oral probiotics were given to regulate intestinal flora, along with vitamin C supplementation and appropriate fluid support therapy. On July 14, 2024, venous blood was collected for microbial blood culture testing at 6:30 a.m. On July 15, 2024, a bone marrow aspiration was performed to rule out hematological malignancies. The results of the bone marrow aspiration showed no abnormal cells or blood parasites, indicating a bone marrow picture consistent with secondary anemia. Other examination results were generally similar to those from the local hospital. On July 18, 2024, the blood culture report indicated that the child tested positive for *Brucella*, and it was discovered that the child’s family raised sheep. By combining clinical symptoms and auxiliary examination results, a diagnosis of brucellosis was confirmed. The treatment plan was adjusted to include the following: (1) Combined intravenous cefotaxime and oral doxycycline for anti-infection. (2) Oral probiotics to regulate intestinal flora. (3) Symptomatic treatment with oral vitamin D and calcium supplements. (4) Nutritional support therapy, including vitamin C supplementation and appropriate fluid replacement. Subsequently, the child’s condition gradually improved. Their general status was good, with no complaints of discomfort, and the child was discharged on 24 July.

## Discussion

*Brucellae* are characterized as small, nonmotile, non-spore-forming, and slow-growing Gram-negative coccobacilli, measuring approximately 0.5–0.7 by 0.6–1.5 µm in size. They belong to the *Brucellaceae* family, which is classified within the alpha-2 subclass of the Proteobacteria phylum. This family also encompasses the genera *Mycoplana, Pseudochrobactrum, Paenochrobactrum, Daeguia, Crabtreella*, and *Ochrobactrum*^[^9^]^.

The clinical manifestations of human brucellosis encompass a wide range of organ systems, typically presenting with nonspecific symptoms such as general malaise, arthralgia, night sweats, and insidious onset of fever. Osteo-articular involvement (including arthritis, sacroiliitis, or spondylitis) and genitourinary complications (such as orchitis, epididymitis, and abortion in pregnant women) are the most frequently observed manifestations^[^10^]^. Cardiovascular complications like endocarditis, myocarditis, pericarditis, or endarteritis are infrequent occurrences (3% of cases). However rare they may be, endocarditis accounts for 80% of deaths among affected individuals^[^11^]^. Aortic and iliac involvement primarily in the form of infected aneurysms is even more uncommon in cases of brucellosis^[^12^]^. Gastrointestinal symptoms are frequently observed in patients with brucellosis and may present as reduced appetite, nausea, vomiting, abdominal discomfort, diarrhea, and constipation. Additionally, hepatosplenomegaly is evident in approximately 50% of individuals affected by brucellosis. In the presence of gastrointestinal disturbances, it is crucial to consider potential complications. Respiratory manifestations of brucellosis are infrequent. They are predominantly documented as clinical cases encompassing pneumonia, pleurisy, pulmonary nodules, pulmonary granuloma, pleural effusion, lung abscess, thoracic abscess, pneumothorax, lymphadenopathy, and mediastinal involvement^[^13,14^]^. Neurobrucellosis is a rare complication of brucellosis, with an incidence ranging from 0.5% to 25% in patients with systemic brucellosis^[^15^]^. The first report of neurobrucellosis dates back to Hughes in 1896. Central nervous system involvement occurs in brucellosis, presenting as a serious and heterogeneous clinical manifestation encompassing encephalitis, meningoencephalitis, radiculitis, myelitis, peripheral and cranial neuropathies, intracranial and subarachnoid hemorrhage, as well as psychiatric manifestations^[^16–18^]^. The prevalence of brucellosis complicated with skin lesions varies between 5% and 17%^[^19^]^. Cutaneous manifestations are nonspecific and include urticaria-like papules and plaques, livedo reticularis, palmar erythema, erythema nodosum^[^20^]^, cutaneous vasculitis^[^21–23^]^, and cutaneous cysts^[^24^]^. Ocular involvement can occur in cases of brucellosis, although such presentations are rare^[^25^]^. All ocular structures may be affected by the disease. Reported cases have demonstrated various ocular and neurological manifestations associated with brucellosis, including uveitis, neuritis, optic neuritis, papilledema, keratitis, and other diverse presentations^[^26^]^. The most frequent manifestation is posterior uveitis^[^27^]^. Thyroiditis caused by *Brucella* has been documented, presenting with typical symptoms of thyroid inflammation, such as fever, chills, and neck swelling^[^28–30^]^.

At present, the detection methods for brucellosis mainly include bacteriological, serological, and molecular biological techniques.

Blood cultures despite the challenges posed by the slow growth characteristics of *Brucella* organisms, laboratory safety concerns, and reduced sensitivity in prolonged disease and focal infections, isolation of the pathogen remains indisputable evidence for confirming the presence of brucellosis. Detection of *Brucella* in blood cultures helps confirm its presence in the disease at an early stage, especially when serological tests still show negative or low or borderline antibody titers^[^31^]^. Furthermore, the isolation of *Brucella* can provide a definitive diagnosis in patients who are not clinically suspected of infection but have been identified as carrying the pathogen in blood cultures obtained from routine examinations^[^32^]^. Currently, the automated blood culture systems predominantly utilized in laboratories include the Bactec 9000 series, Bactec FX series, and BacT/ALERT system developed by Becton Dickinson Company.

Serological diagnosis for brucellosis indirectly confirms exposure to the pathogen by examining the patient’s immune response for antibodies, rather than directly confirming the presence of microorganisms. Currently, serological methods include the Rose–Bangham test (RBT), the tube agglutination test (SAT), which is the most commonly employed serological assay for diagnosing *B. abortus, B. melitensis, and B. suis* infections; the 2-mercaptoethanol test (2-ME); the Coombs antiglobulin agglutination test and the *Brucella* Coombs gel test; the complement fixation test; enzyme-linked immunosorbent assay (ELISA); lateral flow immunochromatography (LFIA), chemiluminescence immunoassay, and time-resolved fluorescence resonance energy transfer detection; as well as fluorescence polarization immunoassay.

Molecular biological detection mainly includes nucleic acid amplification tests (NAATs) and genomic sequencing techniques. Among them, NAATs cover real-time polymerase chain reaction (PCR), quantitative reverse transcription PCR (RT-qPCR), multiplex real-time PCR (M-RT-PCR), and loop-mediated isothermal amplification, among others. Recently, researchers have developed a high-resolution melting-PCR method, which can rapidly screen and directly classify DNA into one of the 12 species within the *Brucella* genus^[^33^]^. Additionally, a droplet digital PCR technology has been established to precisely quantify the *Brucella* DNA load in whole blood samples and evaluate its significance in the diagnosis, prognosis, and treatment of human brucellosis^[^34^]^. Meanwhile, whole-genome sequencing, allele-based typing, and core genome single nucleotide polymorphism techniques have also been widely applied in brucellosis-related research^[^35,36^]^.

The *Brucella* detection technology has achieved certain development to a certain extent. Recently, the combined rapid detection of anti-*Brucella* IgG/IgM can be achieved based on surface-enhanced Raman scattering-LFIA technology^[^37^]^. Researchers have discovered that the novel CRISPR/Cas12a probe can be used to rapidly identify *Brucella* and its species in vitro and on-site^[^38^]^. On the other hand, researchers are committed to identifying novel antigens for the diagnosis of brucellosis. Notable examples include periplasmic protein 26 (BP26)^[^39^]^, multi-epitope fusion proteins^[^40^]^, and various combinations of serum proteins^[^41^]^.

In conclusion, the nonspecific symptoms of brucellosis frequently result in misdiagnosis or delayed diagnosis; however, early intervention can significantly mitigate the risk of complications, shorten the treatment duration, and enhance prognosis. It is advisable to conduct bacterial culture or nucleic acid testing for etiological examination. Serological screening tests should encompass RBT, GICA, or ELISA methodologies. Furthermore, SAT, CFT, or Coombs test may be utilized for serological confirmation. Nevertheless, these diagnostic methods possess certain limitations including time consumption and the potential occurrence of false negatives and false positives as well as relatively low sensitivity. Therefore, it is imperative to develop robust diagnostic assays for brucellosis to improve early detection and treatment outcomes of this disease. Furthermore, healthcare professionals are required to possess a thorough understanding of the clinical manifestations and diagnostic complexities associated with brucellosis in order to ensure accurate and timely diagnoses.

## Data Availability

The original contributions presented in the study are included in the article. For further inquiries, please contact the corresponding authors.
